# A Grape Seed Procyanidin Extract Ameliorates Fructose-Induced Hypertriglyceridemia in Rats via Enhanced Fecal Bile Acid and Cholesterol Excretion and Inhibition of Hepatic Lipogenesis

**DOI:** 10.1371/journal.pone.0140267

**Published:** 2015-10-12

**Authors:** Laura E. Downing, Rebecca M. Heidker, Gianella C. Caiozzi, Brian S. Wong, Kelvin Rodriguez, Fernando Del Rey, Marie-Louise Ricketts

**Affiliations:** Department of Agriculture, Nutrition and Veterinary Sciences, University of Nevada Reno, Reno, Nevada, United States of America; State University of Rio de Janeiro, Biomedical Center, Institute of Biology, BRAZIL

## Abstract

The objective of this study was to determine whether a grape seed procyanidin extract (GSPE) exerts a triglyceride-lowering effect in a hyperlipidemic state using the fructose-fed rat model and to elucidate the underlying molecular mechanisms. Rats were fed either a starch control diet or a diet containing 65% fructose for 8 weeks to induce hypertriglyceridemia. During the 9^th^ week of the study, rats were maintained on their respective diet and administered vehicle or GSPE via oral gavage for 7 days. Fructose increased serum triglyceride levels by 171% after 9 weeks, compared to control, while GSPE administration attenuated this effect, resulting in a 41% decrease. GSPE inhibited hepatic lipogenesis via down-regulation of sterol regulatory element binding protein 1c and stearoyl-CoA desaturase 1 in the fructose-fed animals. GSPE increased fecal bile acid and total lipid excretion, decreased serum bile acid levels and increased the expression of genes involved in cholesterol synthesis. However, bile acid biosynthetic gene expression was not increased in the presence of GSPE and fructose. Serum cholesterol levels remained constant, while hepatic cholesterol levels decreased. GSPE did not modulate expression of genes responsible for esterification or biliary export of the newly synthesized cholesterol, but did increase fecal cholesterol excretion, suggesting that in the presence of GSPE and fructose, the liver may secrete more free cholesterol into the plasma which may then be shunted to the proximal small intestine for direct basolateral to apical secretion and subsequent fecal excretion. Our results demonstrate that GSPE effectively lowers serum triglyceride levels in fructose-fed rats after one week administration. This study provides novel insight into the mechanistic actions of GSPE in treating hypertriglyceridemia and demonstrates that it targets hepatic *de novo* lipogenesis, bile acid homeostasis and non-biliary cholesterol excretion as important mechanisms for reducing hypertriglyceridemia and hepatic lipid accumulation in the presence of fructose.

## Introduction

Rates of obesity, type 2 diabetes, non-alcoholic steatohepatitis (NASH) and non-alcoholic fatty liver disease (NAFLD) have increased significantly in recent years, both in children and adults [1–4]. This surge has correlated with a significant increase in dietary fructose intake in the United States, due in large part to the rise in consumption of sugar-sweetened beverages [5]. Fructose is a highly lipogenic dietary factor [6] and increasing evidence points to an obesogenic role for fructose via the generation of substrates for *de novo* lipogenesis, resulting from rapid hepatic metabolism [7]. Since fructose metabolism is insulin-independent [2], there is less uptake and catabolism of triglyceride (TG)-rich lipoproteins by tissues, ultimately resulting in increased postprandial plasma TG levels [8]. Increased hepatic lipogenesis, combined with decreased uptake of TGs in peripheral tissues, is an important mechanism by which fructose induces steatosis and elevates serum TG levels [9].

In contrast to fructose-induced metabolic dysregulation, evidence indicates that diets rich in fruits and vegetables, e.g. the Mediterranean diet, exert protective effects against the development of the metabolic syndrome [10]. Such diets tend to be high in flavonoids, which exhibit cardioprotective effects in humans [11, 12]. Dietary procyanidins, a class of flavonoids commonly found in grapes, apples and red wine, have been shown to ameliorate risk factors associated with hypertriglyceridemia and steatosis [13–17]. We previously reported that a grape seed procyanidin extract (GSPE) exerts hypotriglyceridemic effects *in vivo* in a normolipidemic state [18–22]. We identified GSPE as a co-agonist ligand for the farnesoid x receptor (nuclear receptor subfamily 1, group H, member 4; *FXR*) [22], a transcription factor that regulates bile acid (BA), TG, cholesterol and glucose homeostasis [23–27]. Mechanistically, GSPE functions in conjunction with BAs, the endogenous ligands of FXR, to upregulate the expression of small heterodimer partner (nuclear receptor subfamily 0, group B, member 2; *Shp*), which represses sterol regulatory element binding protein 1c (*Srebp-1c*) expression, a key regulator of lipogenesis, resulting in the concomitant decrease in downstream lipogenic gene expression [19, 22]. Evidence also shows that GSPE administration increases fatty acid β-oxidation [19] and reduces VLDL-TG secretion [21].

Previous studies utilized a high-fat diet to examine the effects of GSPE [20, 28], however, since sugar intake is also a critical factor that can modulate metabolic homeostasis, particularly TG levels [29, 30], evaluation of the potential therapeutic impact of GSPE on animal models subjected to other dietary regimens, such as carbohydrate-induced hypertriglyceridemia, is warranted. Consequently, the aim of the current study was to more closely mimic a real-world scenario, by assessing whether GSPE can mitigate the effects of existing fructose-induced hypertriglyceridemia *in vivo*, and to determine the underlying molecular mechanisms. This investigation has important implications for further investigations in human subjects using GSPE as a potential natural therapy to counteract increased incidences of hypertriglyceridemia and steatosis.

In the present study, we show that a high-fructose diet for 8 weeks significantly increases serum TG levels in rats, while also markedly inducing hepatic lipid accumulation (steatosis). Co-administration of GSPE with the high-fructose diet for one week only, during the 9^th^ week of the study, effectively ameliorated the adverse consequential effects on serum TGs resulting from the high-fructose diet. This attenuation was achieved via enhanced fecal BA, total lipid, cholesterol and non-esterified fatty acid excretion, inhibition of hepatic *de novo* lipogenesis and increased TG catabolism. We evaluated the gene regulatory effects exerted by GSPE in the liver in the presence of fructose to gain a better insight and understanding regarding the molecular effects leading to the observed hypotriglyceridemic effect.

## Materials and Methods

Grape Seed Procyanidin Extract (GSPE) was obtained from *Les Dérives Résiniques et Terpéniques* (Dax, France). The extract was analyzed in-house using normal phase high performance liquid chromatography (HPLC), as previously described [31] to determine procyanidin composition based on the degree of polymerization. As shown in S1 Fig, GSPE is comprised of procyanidin monomers (68.68 ± 0.02%), dimers (26.16 ± 0.01%) and trimers (5.16 ± 0.02%).

### Animal feeding studies and diets

Rats were housed under standard conditions and all experimental procedures were approved by the local Institutional Committee for Care and Use of Laboratory Animals (IACUC) at the University of Nevada, Reno (Protocol # 00502). Male Wistar rats, 7 weeks of age, were purchased from Charles River Laboratories. After one week of acclimation, rats were randomly assigned to either a control diet (n = 5) or fructose diet (n = 8) for 8 weeks (Harlan Teklad). As shown in Table 1, the starch control diet was a modification of AIN-93G (TD.94045) replacing all sucrose with starch, and was comprised of (% by weight) 17.7% protein, 58.9% carbohydrate and 7.2% fat, providing 3.7 Kcal/g (TD.110787). The fructose diet was a modification of AIN-93G replacing all sucrose and starch with fructose, and was comprised of 17.7% protein, 64.7% carbohydrate and 7.2% fat, providing 3.9 Kcal/g (TD.110786). The animals were maintained on the AIN-93G formulated diets throughout the 9-week study, consistent with previous reports [32, 33]. Food was replenished 3 times per week and food intake was estimated by subtracting the total amount of feed and the amount remaining in the box. Rats were weighed weekly.

**Table 1:** Composition of the diets containing starch (control) and fructose as the major source of carbohydrate^a^.

Ingredient	Control Diet (g/kg)	Fructose Diet (g/kg)
Fructose	0	647
Casein	200	200
L-Cystine	3	3
Corn Starch	515	0
Maltodextrin	132	0
Soybean Oil	70	70
Cellulose	32.486	32.486
Mineral Mix	35	35
Vitamin Mix	10	10
Choline Bitartrate	2.5	2.5
Tert-butylhydroquinone (TBHQ)	0.014	0.014

^a^Formulated and supplied by Harlan Teklad.

Blood samples were collected at 0, 4 and 8 weeks to measure serum triglyceride levels. After 8 weeks on the diets, the rats were randomly assigned to receive either vehicle (water) or GSPE (250 mg/kg) via oral gavage for 7 days, while still consuming their assigned diets. The dose of GSPE used in this study is one-fifth of the no-observed-adverse-effect level (NOAEL) described for GSPE in male rats [34]. This dose is effective in reducing serum TG levels in normolipidemic C57BL/6 mice [19, 22] and rats [18], and was chosen to aid in the identification of the primary, short-term effects of grape seed procyanidins on lipid metabolism in the presence of fructose, in order to gain insight into the mechanisms that underlie their potential longer-term effects. Using a translation of animal to human doses based on metabolic body size [35] and estimating the food intake for a 60-kg human, the dose of GSPE used herein corresponds to ~1.8 g, which is less than a 2 g/day dose previously tested in human subjects [36]. On day 7, the rats were gavaged at 9am, food was then removed and they were sacrificed 5 hours later. Rats were anesthetized using isoflurane, blood was collected from the saphenous vein, after which the animals were euthanized using carbon dioxide, livers were excised and weighed, snap frozen in liquid nitrogen and stored at –80°C. For collection of the feces, rats were placed in clean cages 3 days prior to the end of the experiment and feces were manually collected at the end of the study, air-dried and weighed.

### Gene expression analysis

Total RNA was extracted from liver using TRIzol (Life Technologies) according to the manufacturer’s instructions. Complimentary DNA (cDNA) was reverse transcribed using superscript III reverse transcriptase (Life Technologies), and real-time quantitative polymerase chain reaction (qPCR) was used to determine gene expression changes. qPCR was performed using a CFX96 Real-Time System (BioRad). Forward and reverse primers and probes were designed using Primer3Plus software [37] and purchased from Sigma-Aldrich or Integrated DNA Technologies. Expression of β*-actin* and TATA box binding protein (*Tbp*) were used as endogenous controls. All gene names, abbreviations and accession numbers can be found in S1 Table. Primer and probe sequences can be provided upon request.

### Biochemical Analysis

Serum triglyceride and total cholesterol were measured enzymatically using Infinity^TM^ kits (Thermo Scientific) according to the manufacturers’ instructions. Serum bile acid levels were measured using the Total Bile Acids Assay kit from Diazyme Laboratories, and serum free fatty acids were measured using a non-esterified fatty acid enzymatic colorimetric HR series NEFA-HR (2) assay from WAKO Chemicals USA Inc., performed according to the manufacturer’s instructions. Lipoprotein Lipase (LPL) activity was measured in serum using an ELISA kit (Cell Biolabs, Inc, San Diego, CA), and serum alanine aminotransferase (ALT) and aspartate aminotransferase (AST) were measured using Teco Diagnostic Kits (A526 and A561 respectively), according to the manufacturer’s instructions.

### Histology

After removal from the rat, sections of excised liver were immediately immersed in 10% buffered formalin, and processed for hematoxylin and eosin (H&E) staining, which was performed by the Pathology Laboratory at University of Nevada, Reno.

### Evaluation of Steatosis by Image Analysis

At least three non-consecutive microscopic fields per animal were randomly analyzed, by blindly moving the field of view, using an Olympus DP71 camera attached to a BX41 microscope, equipped with a UPlanFL 40X objective. Each image field was analyzed using a 94x70 point grid (P_T_). The volume density of hepatic steatosis (V_V_ [steatosis, liver]) was then estimated as the ratio of the points marking the vesicles of fat (P_P_) compared to the number of test points using the following equation: V_V_ [steatosis, liver] = P_P_ [steatosis]/P_T_, as previously described [38].

### Hepatic cholesterol measurement

Hepatic cholesterol was extracted using the Folch extraction method and levels were measured as previously described [39, 40]. Briefly, lipids were extracted from 100 mg of liver using a chloroform/methanol mixture. Cholesterol was then determined using a colorimetric Infinity cholesterol assay kit, performed according to the manufacturers’ instructions. Extracted cholesterol contents were normalized to wet liver weight.

### Measurement of fecal bile acid, total lipid, cholesterol and free fatty acid excretion

A modified version of the method reported by Modica et al., was used to measure fecal BA content [40]. Briefly, 0.2 g of dried feces was mixed with 2 ml of 2 mg/ml sodium borohydrate in ethanol and incubated at room temperature for 1 hour. Hydrochloric acid and sodium hydroxide were added and samples were vortexed and allowed to digest for 12 hours under reflux. The samples were then filtered and dried under nitrogen. Samples were re-suspended in milli-Q water and filtered through Sep-Pak C18 cartridges (Thermo Scientific), washed and eluted with methanol and dried under nitrogen. Samples were re-dissolved in 1 ml methanol and BA concentrations were measured enzymatically, using the Total Bile Acids Assay kit from Diazyme Laboratories. Fecal cholesterol and non-esterified fatty acids were extracted as previously described [40], and cholesterol levels were measured using a colorimetric Infinity cholesterol assay kit and non-esterified fatty acids were quantified using a Wako diagnostics HR Series NEFA-HR (2) assay. Total fecal lipids were assessed via gravimetric analysis following Folch extraction. Briefly, 0.5 ml aliquots of the chloroform layer were placed into pre-weighed glass tubes (three tubes per sample) and allowed to evaporate in a fume hood overnight. The next day, the weights were recorded and converted to percent lipids (mg lipid/mg dry fecal weight).

### Statistical Analysis

Data represents the mean ± SEM for the fold change relative to control (or endogenous gene expression) (n = 4 or 5 per treatment, per group, analyzed in triplicate). One-way analysis of variance (ANOVA) followed by Holm-Sidak post-hoc tests was employed to detect significant differences between groups. Treatment differences were considered statistically significant at p<0.05. All statistical analyses were performed using GraphPad Prism version 6.05 for Windows, GraphPad Software (San Diego, CA).

## Results

### Effect of fructose feeding for 8 weeks and co-administration with GSPE for the 9^th^ week of the study on body weight, liver weight and serum biochemical analysis

Fructose feeding for 8 weeks did not significantly increase body weight compared to the rats fed the control diet (Table 2). As shown in S2A Fig, the fructose fed rats tended to have a lower body weight compared to the control animals after the first week on the diets, a trend which continued through to week 8. Food intake was not statistically different between the groups.

**Table 2:** Body and liver weight and % liver weight to body weight ratio at the time of sacrifice^a^.

	Control-VEH	Fructose-VEH	Fructose-GSPE
Body Weight (g)	509.04 ± 9.56	461.25 ± 15.23*	468.45 ± 8.38*
Liver Weight (g)	14.94 ± 0.32	15.70 ± 0.91	15.75 ± 0.75
% liver weight to body weight ratio	2.94 ± 0.08	3.40 ± 0.11**	3.36 ± 0.10*

^a^Rats were fed either the control or fructose diet for 8 weeks. Vehicle (water) or GSPE (250 mg/kg) was administered daily via oral gavage during the 9^th^ week of the study.

Significantly different from control:

*p<0.05

**p<0.01.

GSPE administration during the 9^th^ week of the study did not have any significant effect on the body weight of the rats in the fructose-GSPE group, compared to the fructose-vehicle group. As shown in S2B Fig, the group of animals assigned to receive GSPE in week 9 demonstrated no significant differences in body weight during the course of the study. In contrast, the fructose-fed rats had a significantly higher liver to body weight ratio compared to the control group at the end of the study, as shown in Table 2, with no significant differences observed following GSPE administration.

**Fig 1:**
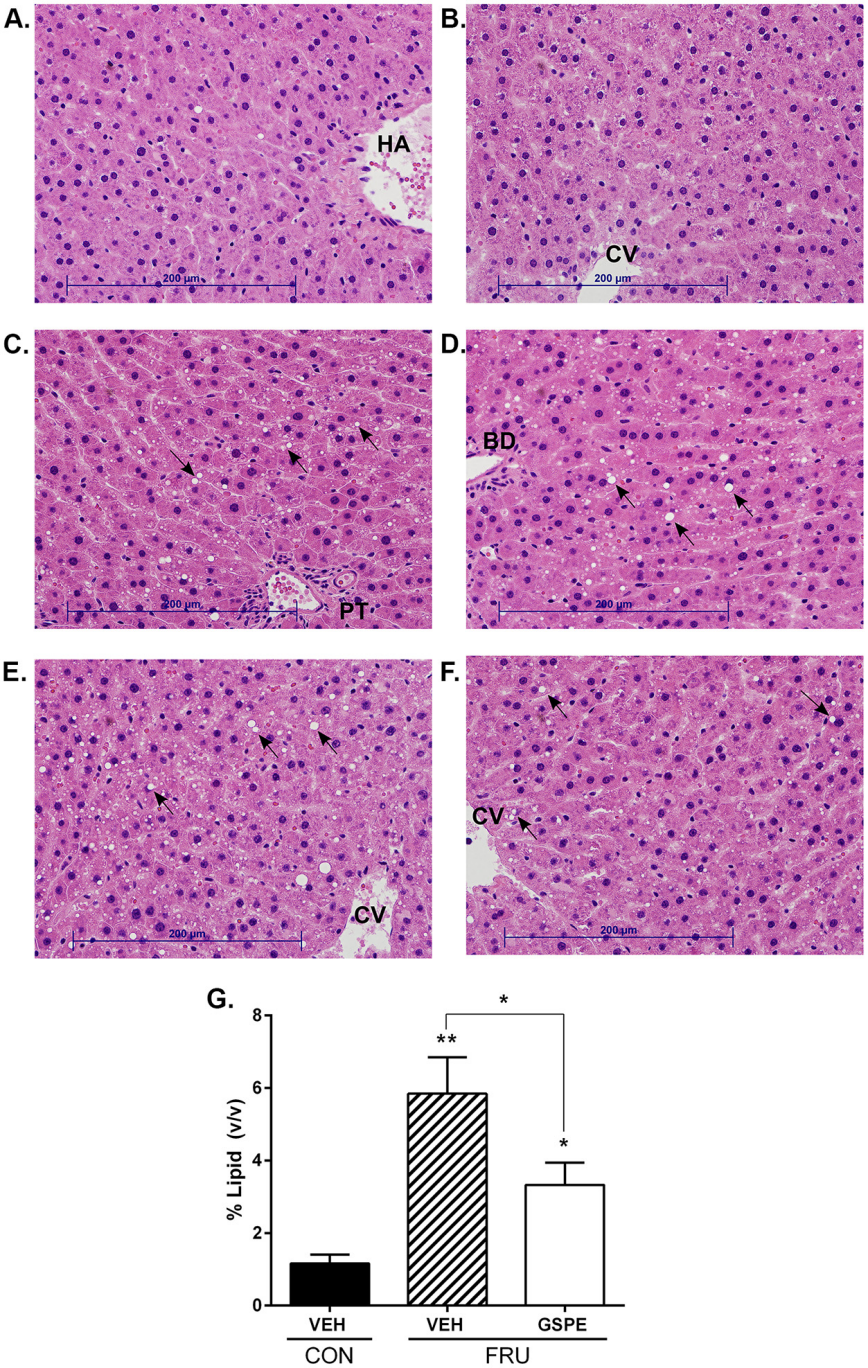
Fructose-induced hepatic lipid accumulation is ameliorated by GSPE administration. Representative liver histology sections stained with hematoxylin and eosin from (**A** and **B**) Control, (**C** and **D**) Fructose-vehicle and (**E** and **F**) Fructose-GSPE treated rats. Lipid droplet infiltration is evident in the fructose-fed rats as indicated by the black arrows (**BD**: Bile duct; **CV**: Central vein; **HA**: Hepatic artery; **PT**: Portal triad); **(G)** Lipid accumulation induced by fructose consumption was evaluated using image analysis of the histological sections from each animal. The volume of lipid droplets was assessed as a percentage of the total hepatic volume. *p<0.05 and **p<0.01.

**Fig 2:**
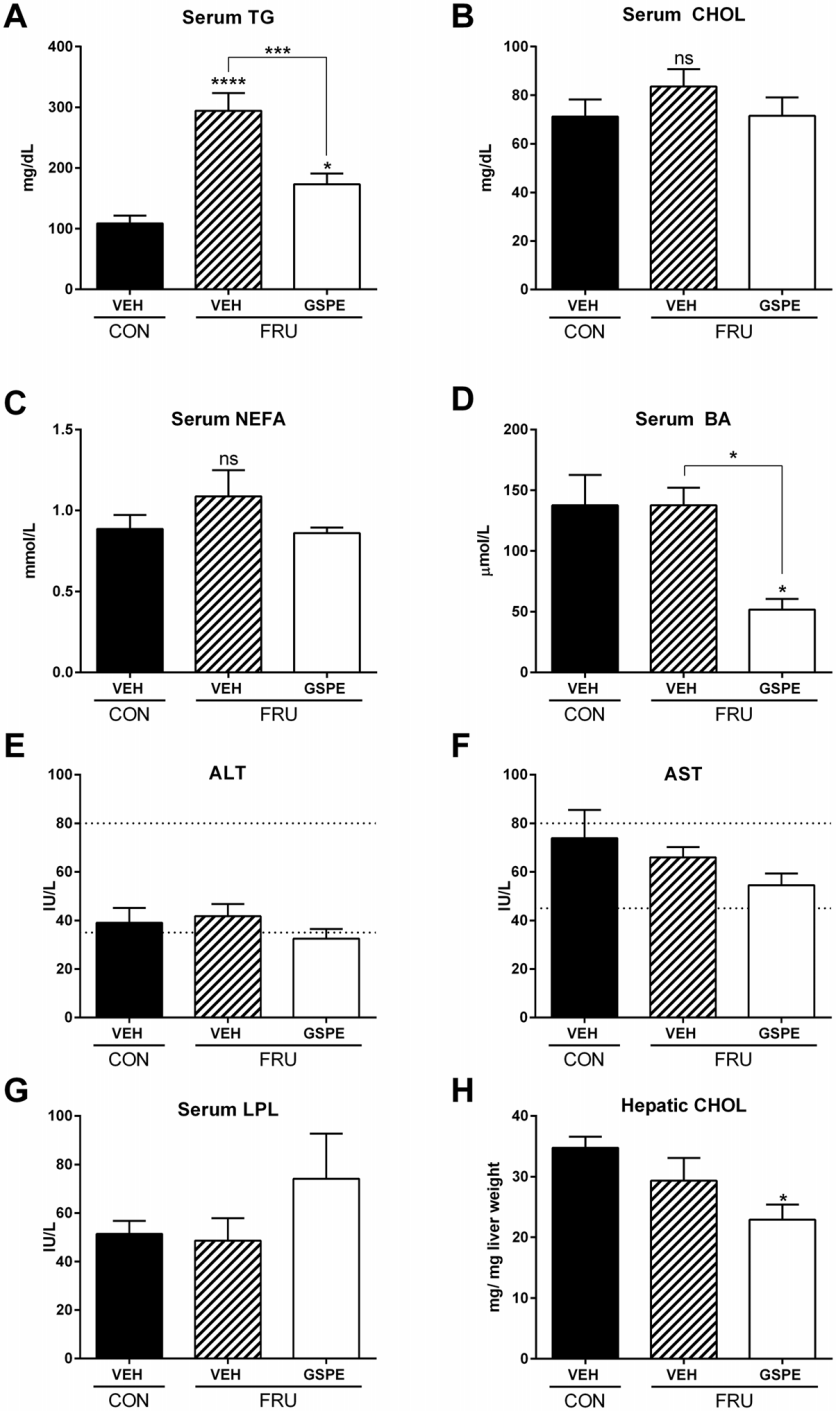
Serum biochemical analysis and hepatic cholesterol content. Serum was analyzed for (**A**) triglycerides (TG) (**B**) cholesterol (CHOL), (**C**) non-esterified fatty acids (NEFA), (**D**) bile acids (BA), (**E**) Alanine aminotransferase (ALT), (**F**) aspartate aminotransferase (AST); the dotted lines represent the normal upper and lower limits respectively. (**G**) lipoprotein lipase activity (LPL), and (**H**) hepatic cholesterol content (CHOL). *p<0.05, ***p<0.001 and ****p<0.0001.

**Fig 3:**
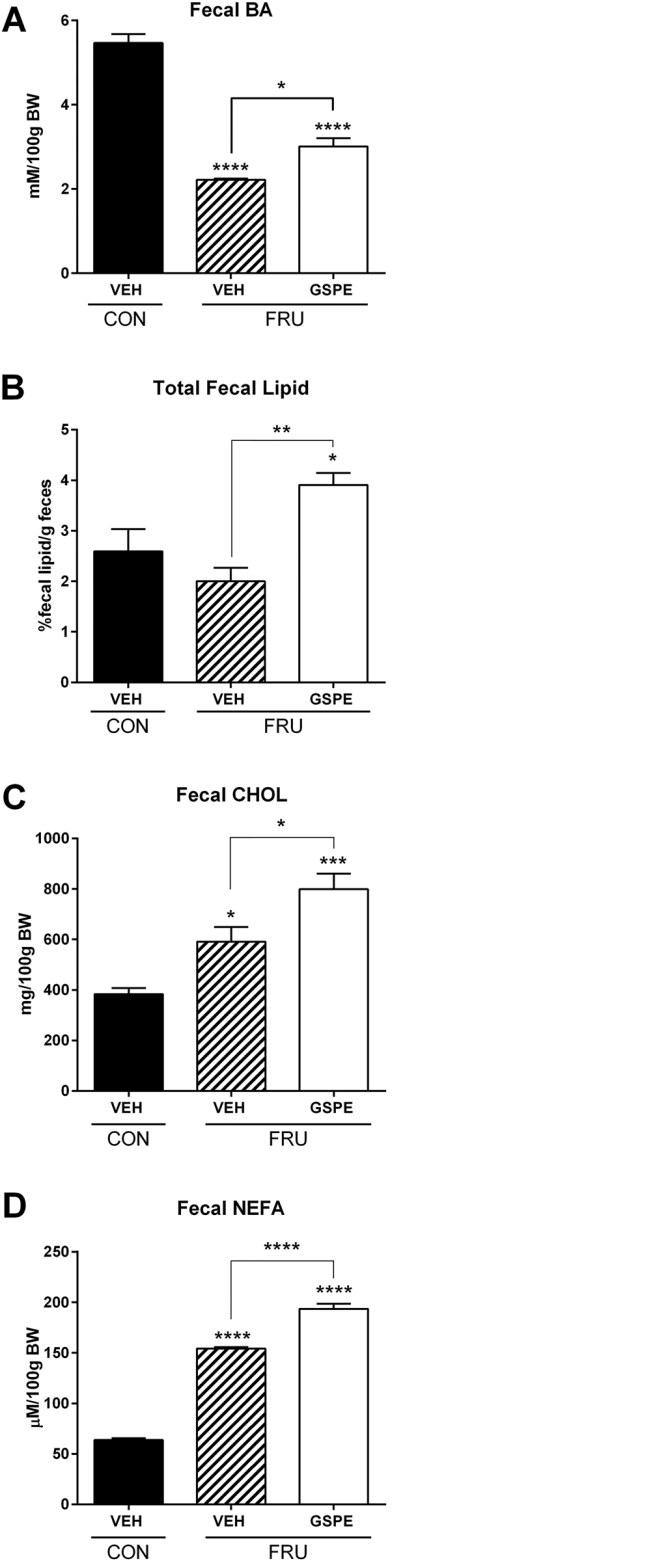
GSPE increases fecal bile acid, total fecal lipid, cholesterol and fatty acid excretion. Fecal (**A**) bile acids (BA), (**B**) total fecal lipid, (**C**) cholesterol (CHOL) or (**D**) non-esterified fatty acids (NEFA) were analyzed. *p<0.05, **p<0.01, ***p<0.001 and ****p<0.0001.

Consistent with the increased liver weight, fructose-fed animals had a significantly higher grade of microvesicular steatosis and increased hepatic lipid accumulation volume density, compared to the control group (Fig 1). The fructose-fed rats also showed evidence of mild inflammatory infiltration around the portal triad (Fig 1C). GSPE administration for one week resulted in a significant decrease in hepatic lipid volume density and therefore steatosis (Fig 1E, 1F and 1G). The fructose diet increased serum TG levels by 171% compared to the control group after 9 weeks (Fig 2A), which was ameliorated by treatment with GSPE resulting in a 41% reduction. No significant changes were observed in the fructose-fed rats with respect to serum cholesterol (Fig 2B) or NEFA levels (Fig 2C). Serum BA levels were not altered by the fructose diet alone, however, co-administration with GSPE for one week significantly reduced serum BA levels (Fig 2D). Neither fructose feeding nor GSPE administration caused liver damage, as evidenced by the fact that the values for both ALT and AST remained within normal limits (Fig 2E and 2F). Lipoprotein lipase activity did not change upon fructose consumption, nor following GSPE administration (Fig 2G). Hepatic cholesterol levels were significantly reduced following GSPE administration (Fig 2H). Fecal BA levels were significantly decreased by the fructose diet, and increased following GSPE administration (Fig 3A). Total fecal lipid excretion was significantly increased by GSPE in the fructose-fed animals (Fig 3B). Fecal cholesterol (Fig 3C) and NEFA (Fig 3D) were both increased by the fructose diet, while GSPE further enhanced their excretion.

### Expression of genes involved in hepatic lipogenesis, cholesterol & bile acid synthesis and transport

**Fig 4:**
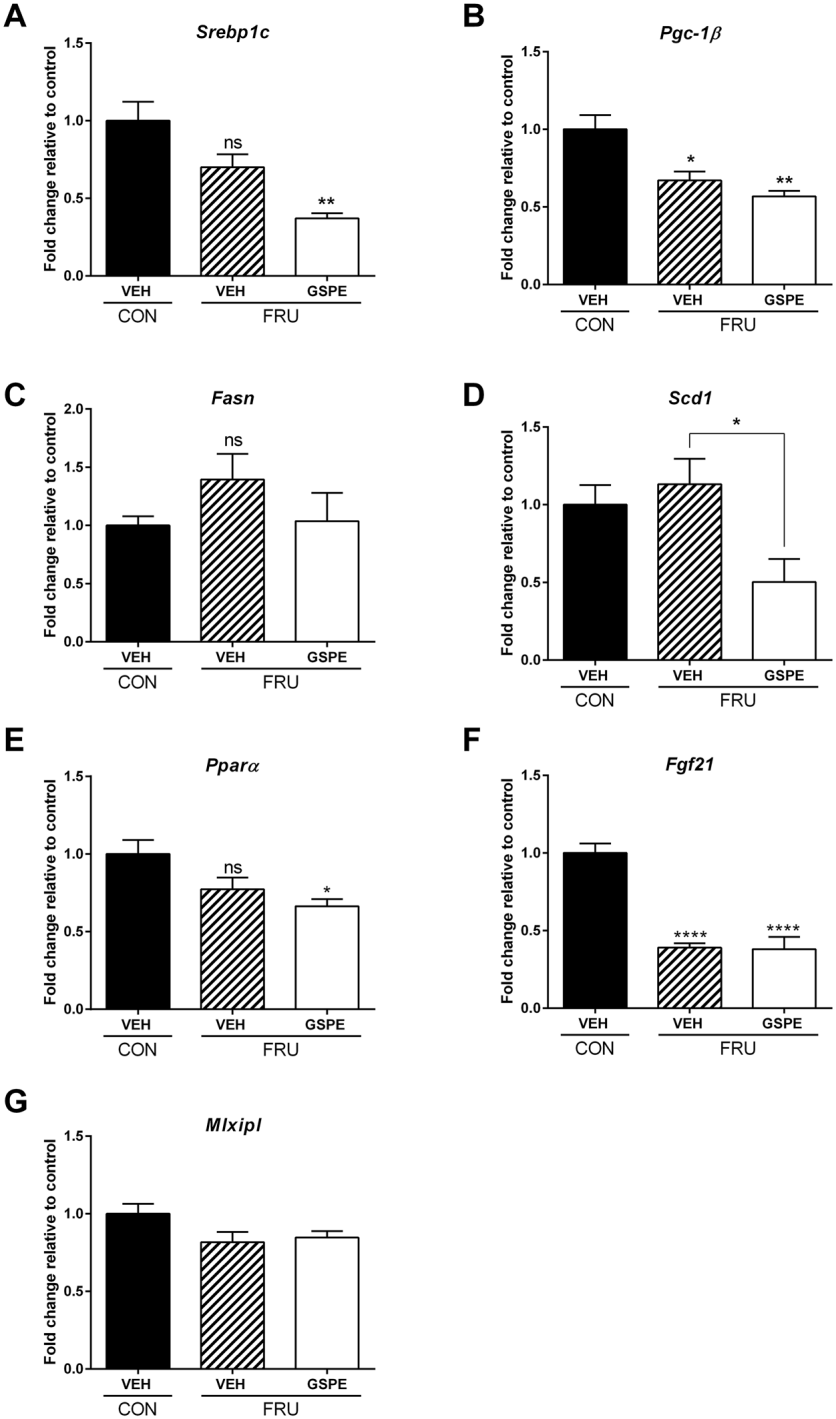
Hepatic expression of genes involved in lipogenesis. Gene expression changes were analyzed for (**A**) *Srebp-1c*, (**B**) *Pgc-1β*, (**C**) *Fasn*, (**D**) *Scd1*, (**E**) *Pparα*, (**F**) *Fgf21* and (**G**) *Mlxipl*. *p<0.05, **p<0.01 and ****p<0.0001.

To explore the molecular mechanism underlying the hypotriglyceridemic effect of GSPE observed in the fructose-fed animals, we next examined the expression of hepatic genes that regulate lipogenesis, cholesterol synthesis and transport, and BA synthesis and transport. Dietary fructose did not alter sterol regulatory element binding protein 1c (*Srebp-1c*) expression in this study; however, GSPE significantly reduced expression (Fig 4A). Peroxisome proliferator-activated receptor gamma, coactivator 1 beta (*Pgc-1β* activates the expression of genes involved in lipogenesis and TG secretion via direct co-activation of Srebp [41]. Consequently, Pgc-1β, a regulator of both carbohydrate and lipid metabolism, has been proposed to play a pivotal role in fructose-induced lipogenesis [41]. However, fructose feeding reduced *Pgc-1β* expression, compared to the control diet, in this study (Fig 4B). Fructose feeding, either with or without GSPE, had no effect on fatty acid synthase (*Fasn*) expression, a Srebp-1c lipogenic target gene (Fig 4C). Stearoyl-CoA desaturase (delta-9-desaturase) 1 (*Scd1*) another SREBP-1c target gene, although not affected by fructose, was markedly repressed by GSPE (Fig 4D).

An alternative mechanism by which fructose can increase lipogenesis is via repression of peroxisome proliferator-activated receptor alpha (Pparα) [42]. Consequently, we also assessed the effects of fructose feeding on the expression of *Pparα* and fibroblast growth factor 21 (*Fgf21*), a down-stream target of Pparα [43–45]. As shown in Fig 4E, fructose feeding did not alter the expression of *Pparα*, however, *Fgf21* expression was significantly repressed (Fig 4F). *Pparα* expression was significantly repressed by GSPE, compared to the control, while no difference was seen with respect to *Fgf21* expression in the presence of GSPE, compared to fructose only. No significant changes in the expression of MLX interacting protein-like (*Mlxipl*), also referred to as carbohydrate response element binding protein, were observed following fructose ingestion or GSPE administration in this study (Fig 4G).

**Fig 5:**
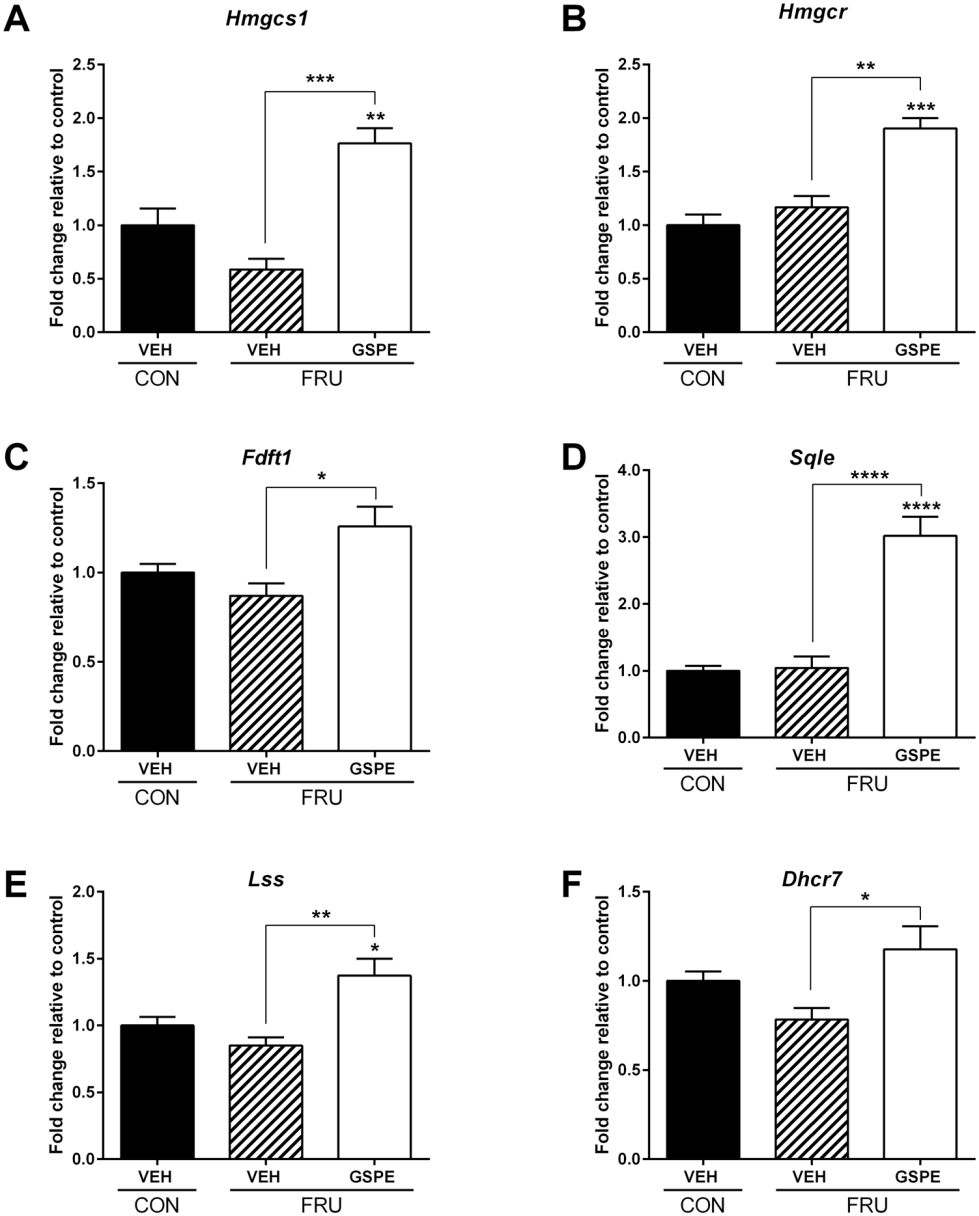
Hepatic expression of genes involved in cholesterol synthesis following treatments. Gene expression changes were analyzed for (**A**) *Hmgcs1*, (**B**) *Hmgcr*, (**C**) *Fdft1*, (**D**) *Sqle*, (**E**) *Lss*, and (**F**) *Dhcr7*. *p<0.05, **p<0.01, ***p<0.001 and ****p<0.0001.

**Fig 6:**
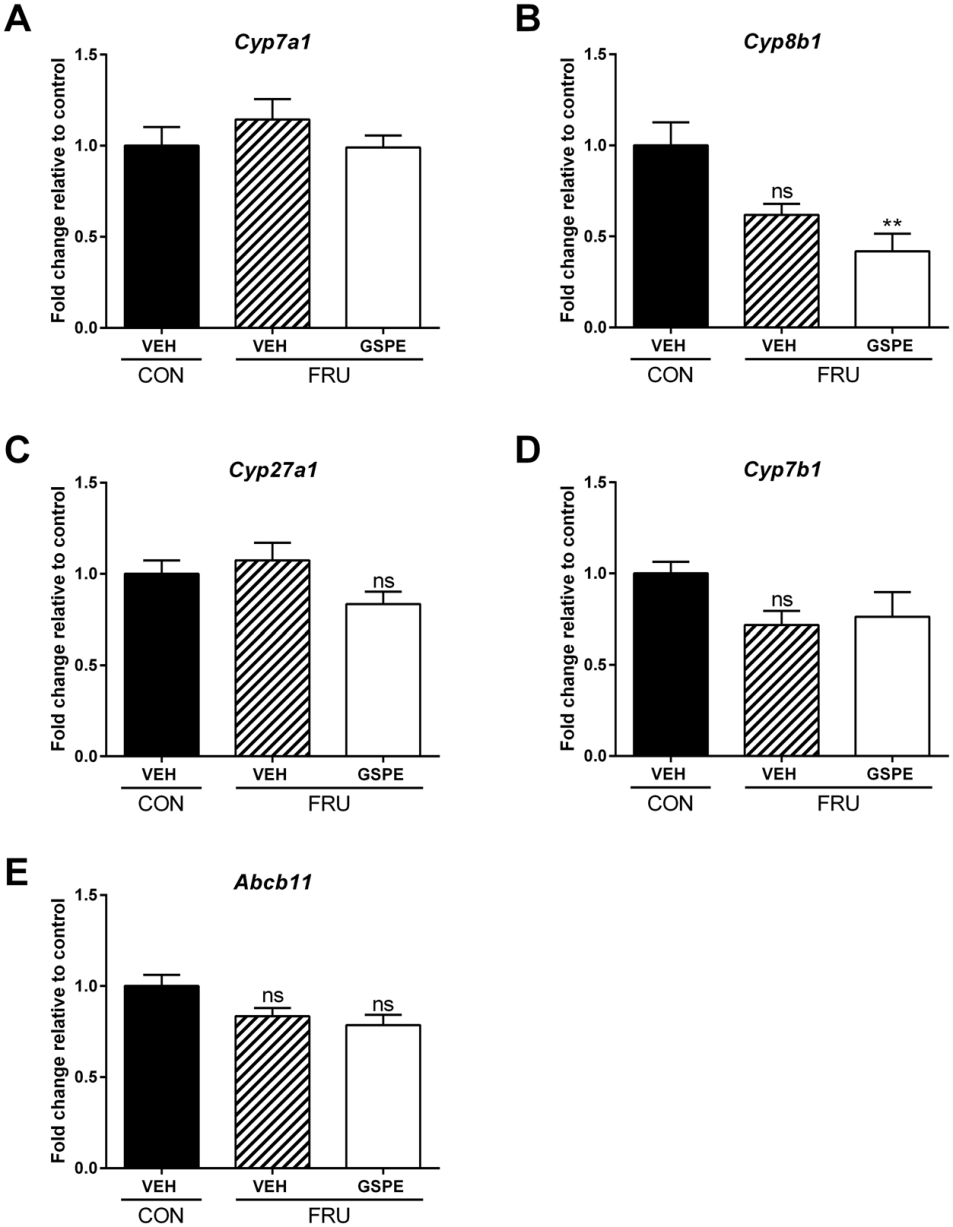
Hepatic expression of genes involved in bile acid biosynthesis and transport following treatments. Gene expression changes were analyzed for (**A**) *Cyp7a1*, (**B**) *Cyp8b1*, (**C**) *Cyp27a1*, (**D**) *Cyp7b1*, and (**E**) *Abcb11*. ** p<0.01.

**Fig 7:**
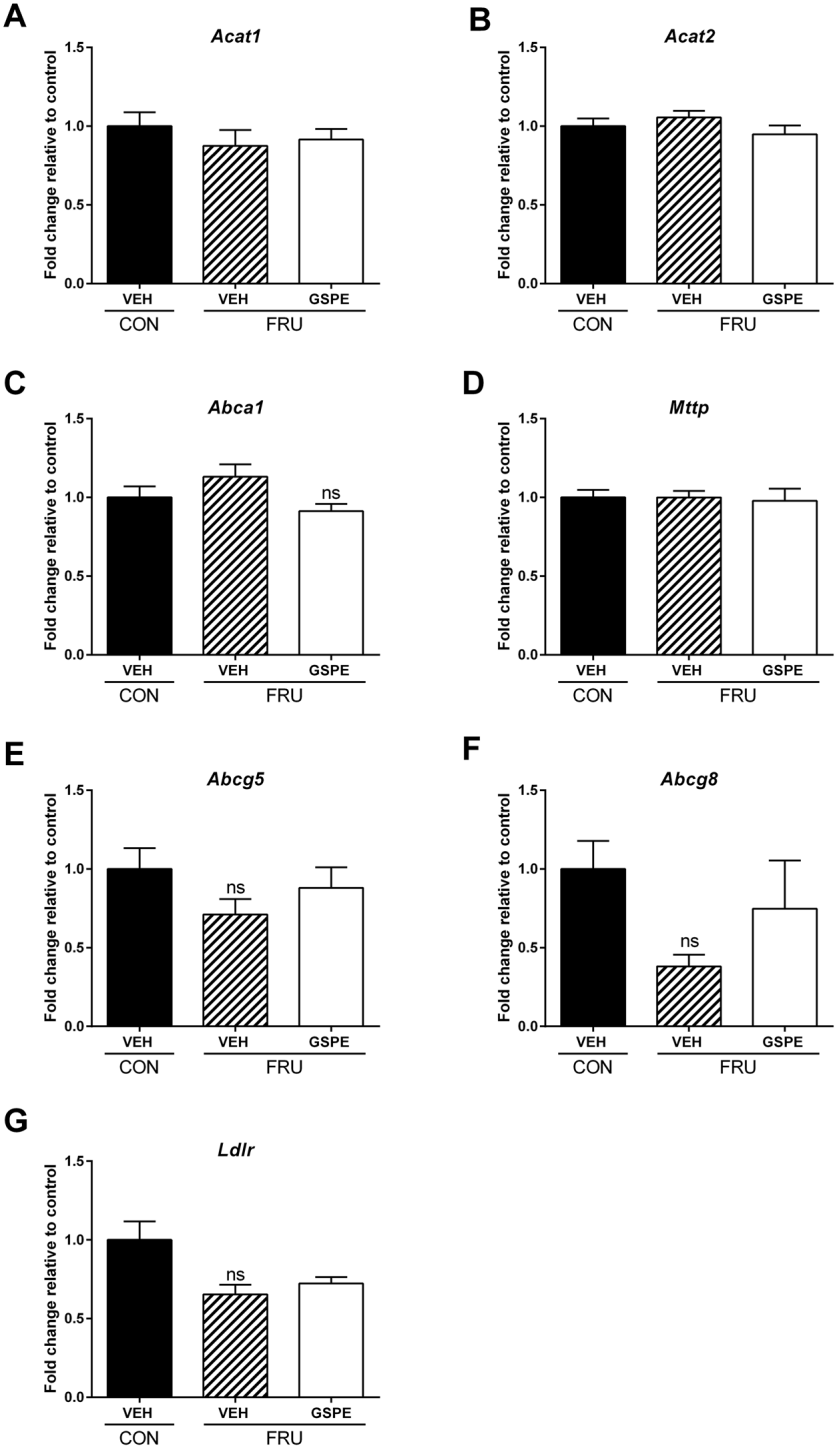
Hepatic expression of genes involved in hepatic cholesterol export. Gene expression changes were analyzed for (**A**) *Acat1*, (**B**) *Acat2*, (**C**) *Abca1*, (**D**) *Mttp*, (**E**) *Abcg5*, (**F**) *Abcg8*, and (**G**) *Ldlr*.

Based on the combined observations of reduced serum TG and BA levels and increased fecal BA excretion, we postulated that serum TG levels were reduced due to the need to synthesize cholesterol and then BAs. Therefore, we next examined potential regulatory effects on genes involved in both cholesterol and BA synthesis. Dietary fructose had no effect on the expression of 3-hydroxy-3-methylglutaryl-CoA synthase 1 (*Hmgcs1*), while GSPE significantly increased expression (Fig 5A). In addition, no changes in expression were seen in the fructose-fed animals with respect to 3-hydroxy-3-methylglutaryl-CoA reductase (*Hmgcr*) (Fig 5B). However, GSPE increased expression of *Hmgcr* compared to both the control and fructose-vehicle-treated animals. In addition, several genes important in cholesterol synthesis were significantly upregulated in the presence of GSPE, including farnesyl-diphosphate farnesyltransferase 1 (*Fdft1*) (Fig 5C), squalene epoxidase (*Sqle*) (Fig 5D), lanosterol synthase (2,3-oxidosqualene-lanosterol cyclase) (*Lss*) (Fig 5E) and 7-dehydrocholesterol reductase (*Dhcr7*) (Fig 5F), indicating that there was increased cholesterol synthesis in the presence of GSPE. Consequently, we next evaluated the effects of GSPE on BA biosynthesis. No significant effects were seen with respect to cytochrome P450, family 7, subfamily A, polypeptide 1, (cholesterol 7 alpha-monooxygenase; *Cyp7a1*) expression, which initiates the *classical (neutral)* BA biosynthetic pathway, following GSPE administration (Fig 6A), while cytochrome P450, family 8, subfamily B, polypeptide 1, (sterol 12-alpha-hydroxylase; *Cyp8b1*) levels were significantly reduced (Fig 6B). We then assessed the effects of GSPE on genes involved in the *alternative (acidic)* BA biosynthesis pathway. No significant effects were observed with respect to cytochrome P450, family 27, subfamily A, polypeptide 1 (steroid 27-hydroxylase; *Cyp27a*1) (Fig 6C) or cytochrome P450, family 7, subfamily B, polypeptide 1 (oxysterol 7-alpha-hydroxylase; *Cyp7b1*) (Fig 6D). In addition, no changes were observed in the expression of ATP-binding cassette, sub-family B (MDR/TAP), member 11 (bile salt export pump; *Abcb11*) (Fig 6E).

Since we observed an increase in endogenous cholesterol synthesis but no changes in the expression of genes regulating BA biosynthesis, despite reduced hepatic cholesterol levels, we next analyzed expression levels of acetyl-CoA acetyltransferase 1 (A*cat*1) and acetyl-CoA acetyltransferase 2 (*Acat2*) to determine whether the newly synthesized cholesterol was being esterified and exported, for assembly into lipoproteins. No changes in *Acat1* (Fig 7A) or *Acat2* expression (Fig 7B) were observed. ATP-binding cassette, subfamily (ABC1), member 1 (*Abca1*) either directly or indirectly mediates the transport of cholesterol and phospholipids across cell membranes, where they are removed from cells by apolipoproteins [46]. We therefore measured *Abca1* and found no changes in expression (Fig 7C). Our previous studies showed that GSPE reduced levels of apolipoprotein B (ApoB) in HepG2 cells [19], and measurement of microsomal triglyceride transfer protein (*Mttp*) expression, which helps to deliver TG to ApoB was not affected by either fructose feeding or GSPE administration in this study (Fig 7D). We next analyzed the expression of ATP-binding cassette, subfamily G (WHITE), member 5 (*Abcg5*) and ATP-binding cassette, subfamily G (WHITE), member 8 (*Abcg8*) to determine whether the unesterified cholesterol underwent biliary export. No significant changes in expression were observed for either *Abcg5* or *Abcg8* (Fig 7E and 7F), and fructose feeding did not alter low density lipoprotein receptor (*Ldlr*) expression (Fig 7G).

## Discussion

The study presented herein provides new evidence establishing that one week administration with GSPE effectively lowers serum TG levels in a model of fructose-induced hypertriglyceridemia. Previous reports showed that GSPE decreases serum TGs in a normolipidemic state in several animal models, including rats [18, 19, 22]. We now show that, under conditions of severe hypertriglyceridemia, GSPE causes a significant 41% reduction in serum TG levels. This is comparable to that seen with fenofibrate, one of the most commonly prescribed lipid-lowering agents in the world [47], which reduces serum TGs by 36% [48]. Cholesterol synthesis gene expression was increased in the fructose-GSPE treated animals, and was accompanied by a concomitant increase in fecal excretion of cholesterol, which could occur via *transintestinal cholesterol efflux* (TICE) [49]. Additionally, compared to the control diet, fructose caused a marked increase in hepatic lipid droplet accumulation, indicative of steatosis (>5% lipid volume), which was significantly attenuated following co-administration with GSPE.

It is well established that high-fructose feeding causes diet-induced alterations in lipid metabolism [50]. In the present study, we observed a significant 171% increase in serum TGs in the rats after 9 weeks on the fructose diet. Increased expression of *Srebp-1c* was previously shown to be one mechanism underlying fructose-induced hypertriglyceridemia, however, it is not the only mechanism involved [8]. Pparα serves as an essential regulator of lipid metabolism, with gene ablation disrupting normal lipid homeostasis [51]. Hepatic suppression of Pparα was also identified as a mechanism contributing to serum hypertriglyceridemia induced by a high-fructose diet [50]. Additionally, reduced Fgf21 levels have been shown to decrease lipolysis [43].

In the current study, GSPE administration in the fructose-fed rats significantly decreased the expression of both *Srebp1c* and *Scd1*, indicating decreased TG synthesis, thereby contributing to reduced serum TG levels. We did not observe an increase in *Srebp-1c* following fructose consumption, likely due to the fact that the rats were fasted for 5 hours prior to sacrifice. It is well known that *Srebp-1c* is sensitively suppressed by fasting or nutritional deprivation [52, 53]. Therefore, fasting could have resulted in the lack of induction in the expression of *Srebp-1c* and its downstream targets, including *Pgc-1β* and *Fasn*, in addition to *Mlxipl*, in the fructose-fed animals. Despite no effect on *Srebp1c*, *Fgf21* expression was reduced in the fructose group, indicating decreased lipolysis [43]. Therefore, it is possible that repression of *Fgf21* is the underlying mechanism by which fructose induced hypertriglyceridemia in these rats.

Reduced serum BA levels observed in the fructose-GSPE treated rats correlate with increased fecal BA output. Indeed, decreased intestinal BA absorption, combined with reduced hepatic lipogenesis, may be linked to the observed reduction in serum TG levels. Reduced BA absorption necessitates increased conversion of cholesterol into BAs. Since the rats consumed a cholesterol-free diet, increased endogenous cholesterol synthesis is necessary for the production of BAs. This is consistent with the observed increase in cholesterol synthesis gene expression observed in this study. Interestingly, there was no effect on *Cyp7a1*, which encodes cholesterol 7α-hydroxylase, the rate-limiting enzyme in BA biosynthesis. *Cyp8b1*, responsible for canonical BA synthesis, was decreased, with no changes in alternative BA biosynthetic gene expression, indicating that the newly synthesized cholesterol is not then shuttled into the pathway for BA production. In addition, the most readily available source of acetyl CoA for use in cholesterol synthesis would be from catabolized TGs, which provides an elegant explanation for the observed reduction in serum TGs, even in the presence of fructose.

GSPE administration profoundly increased total fecal lipid excretion, as well as BA excretion. It is known that hepatobiliary cholesterol excretion is not the only way to remove cholesterol from the body. The proximal part of the small intestine is now known to actively secrete cholesterol, via a pathway called *transintestinal cholesterol efflux* (TICE) [49]. The rate of TICE strongly depends on the presence of a cholesterol acceptor, and increased levels of BAs in the intestinal lumen are known to increase TICE [54]. When bile salts are combined with phospholipid, the TICE pathway is strongly stimulated [55]. Therefore, increased levels of both BA and total lipid within the intestine could stimulate TICE and therefore contribute to enhanced fecal cholesterol excretion. Consequently, serum cholesterol levels remained the same due to an equilibrium being achieved between the rate of endogenous cholesterol synthesis and the amount secreted from the liver into the blood to be subsequently excreted via TICE. Importantly, and in agreement with this notion, the amount of cholesterol excreted in the feces exceeds the levels that could have originated from dietary intake.

In addition to decreased serum TG levels, GSPE treatment significantly decreased hepatic steatosis induced by fructose, as evidenced by the reduction in hepatic lipid droplet accumulation and cholesterol content. Steatosis by itself is considered to be a relatively benign and reversible condition [56]. However, transition from steatosis into NASH represents a key step in pathogenesis, since it sets the stage for further liver damage, including development of fibrosis, cirrhosis and eventually hepatocellular carcinoma [56]. Progression from simple steatosis to NASH usually involves a *“second hit”*, e.g. oxidative stress and inflammation, and previous studies have shown that accumulation of cholesterol, rather than fatty acids or triglycerides, is critical for this progression [57]. Reduction in hepatic cholesterol content has been proposed as a fundamental treatment strategy for NAFLD [58]. It may be speculated that one reason why the newly synthesized cholesterol is not converted to BAs, esterified or moved to the plasma membrane for export via the apolipoprotein/Abca1 pathway by GSPE in the presence of fructose, is because it is removed from the liver as a protective mechanism against further progression of steatosis.

In conclusion, this study provides valuable and innovative insight into the molecular regulatory actions of GSPE in treating hypertriglyceridemia in the fructose-fed rat model. This is achieved at the molecular level via GSPE-induced down-regulation in the hepatic expression of *Srebp1c* and *Scd1*, to decrease lipogenesis, combined with increased endogenous hepatic cholesterol synthesis, without any corresponding increase in *de novo* BA biosynthesis from the newly synthesized cholesterol in the fructose-fed rats. The novel observations resulting from this study demonstrate that, in the presence of fructose, GSPE alters the conversion of endogenously synthesized cholesterol, directing it through TICE for export via the feces. These results, combined with the decreased levels of steatosis, indicate that GSPE warrants further investigation as a treatment strategy against metabolic dysregulation and potential for amelioration of hepatic steatosis.

## Supporting Information

S1 Fig HPLC analysis of the procyanidin composition in grape seed procyanidin extract (GSPE). (TIF)

S2 Fig Body Weight during the 8 week feeding study for (A) Control versus Fructose-fed rats, and (B) Fructose-Vehicle versus Fructose-GSPE treated rats. (TIF)

S1 Table Gene names, abbreviations and accession numbers and synonyms. (TIF)

## Abbreviations

BA: Bile acid
GSPE: Grape seed procyanidin extract
TG: Triglyceride
