# Supplementary figures and images for: A Grape Seed Procyanidin Extract Ameliorates Fructose-Induced Hypertriglyceridemia in Rats via Enhanced Fecal Bile Acid and Cholesterol Excretion and Inhibition of Hepatic Lipogenesis
Source: PLoS One. 2015 Oct 12;10(10):e0140267. doi: 10.1371/journal.pone.0140267 (PMC4601771; doi:10.1371/journal.pone.0140267)

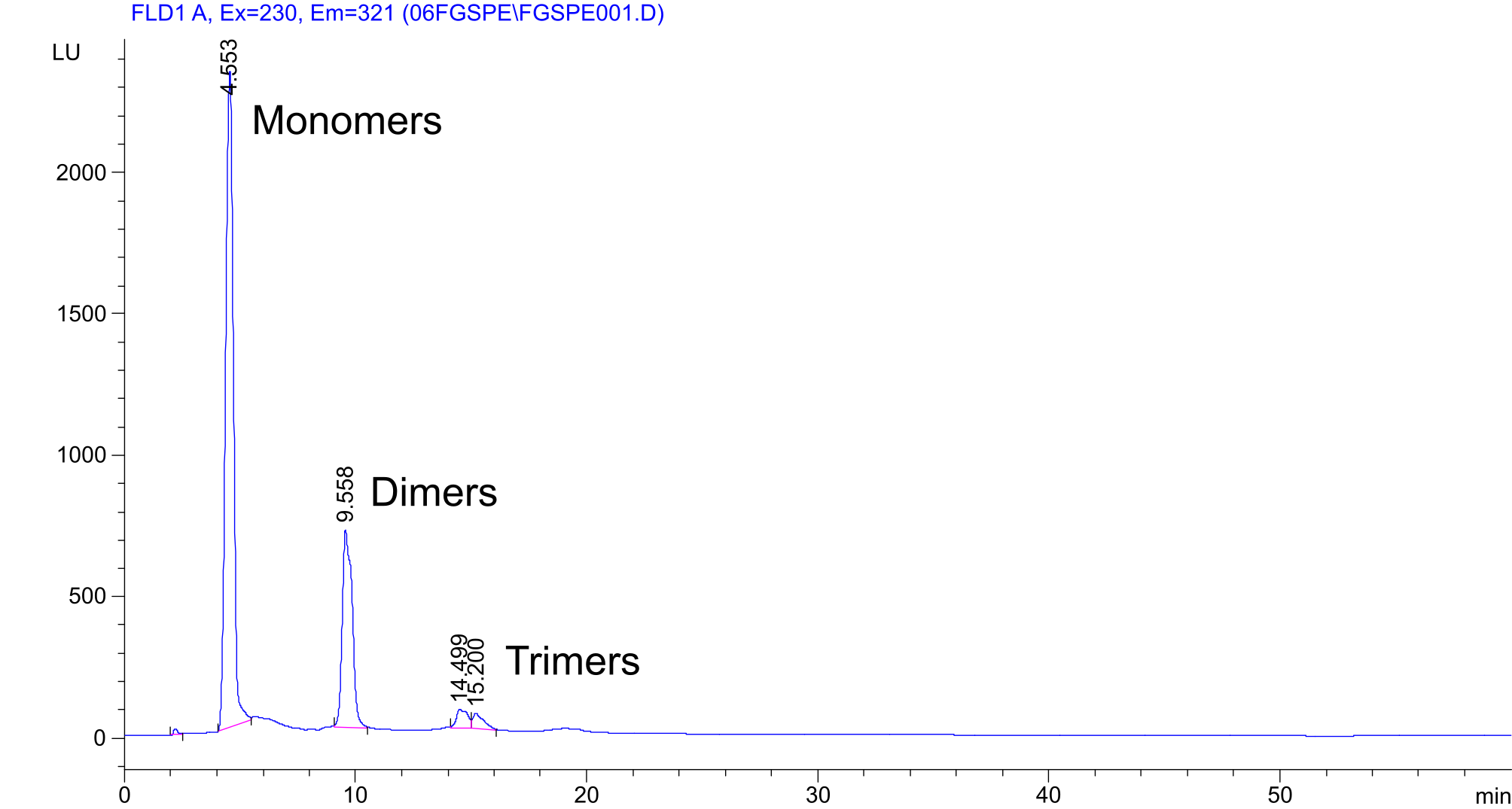

Supplement: S1 Fig — (TIF) [file pone.0140267.s001.tif]

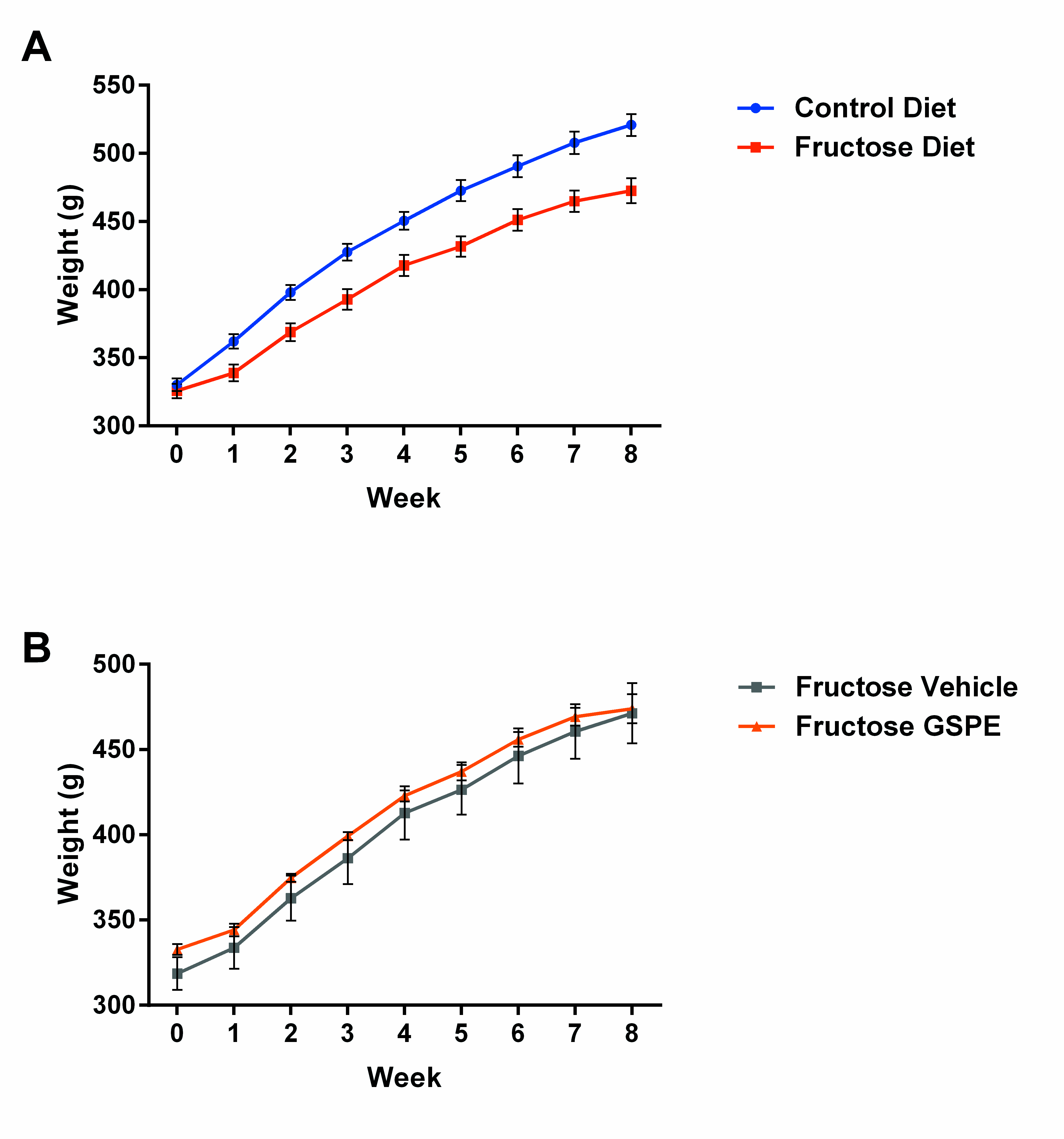

Supplement: S2 Fig — (TIF) [file pone.0140267.s002.tif]

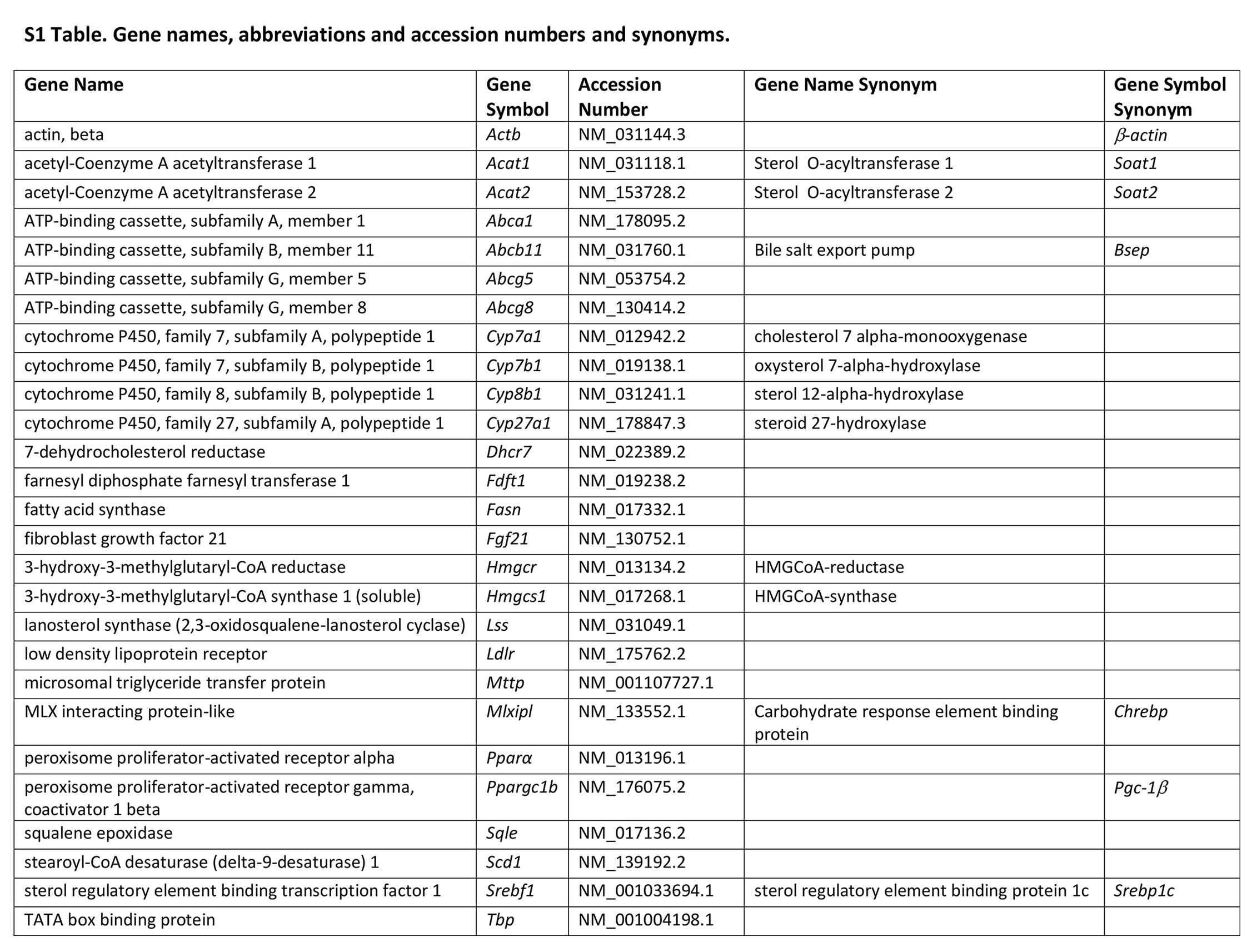

Supplement: S1 Table — (TIF) [file pone.0140267.s003.tif]
